# Supplementary material for: Prevalence and associated factors of diabetes among adult populations of Hawassa town, southern Ethiopia: A community based cross-sectional study
Source: PLoS One. 2025 Jan 30;20(1):e0318081. doi: 10.1371/journal.pone.0318081 (PMC11781642; doi:10.1371/journal.pone.0318081)
Supplement: Attachment — Submitted filename: Response to reviewers.docx [file pone.0318081.s001.docx]

**Dear Editor**

We appreciate you looking over our manuscript and providing us with this valuable input. We made changes to the manuscript in response to the comments, and one of the attached manuscript files shows the highlighted changes. Point by point, we offer our answer, the questions, and our response as follows:

**Comments on Journal requirements:**

**Title page**

**Q1.** Please! Remove the prefix: prof, from HL Sithole
It is my belief that the primary author possesses two addresses, therefore it is imperative that both are included on the title page in the following manner:
Getu Kassa Belete1, 2*, HL Sithole2
1World Health organization (WHO), Sidama, Ethiopia
2College of Health Studies at the University of South Africa (UNISA)
*Corresponding Author
E-mail: [getish98@gmail.com](mailto:getish98@gmail.com)

**Answer for Q1**. *Thank you for this important comments. We have removed the prefix and also indicated the two addresses per comment on page 1.*

**Abstract part**

**Q2.** CI, AOR, DM: Please! Describe fully prior to abbreviating the words

**Answer for Q2.** *We appreciate your feedback. Every acronym was fully explained per the comments on page 2 & 3.*

**Q3.** Being illiterate: it is better, if you say “unable to read and write” than saying “illiterate”

**Answer for Q3.** *Thank you for the comment. As per the comment changed illiterate to unable to read and write on page 2,14,18 and 19.*

**Q4.** Keywords: please! Remove the second keywords “Key words: Magnitude, diabetes, non-communicable, risk factors”

**Answer for Q4.** *Thank you for the feedback. We removed the second keywords per the comment on page 3.*

**Q5.** Please! Use similar writing manner when writing: medium cycle (AOR: 2.79; 95% CI: 1.02, 7.63), having ever chewed khat (AOR: 6.50; 95% CI: 4.07, 10.39)

**Answer for Q5**. *Thank you for the comment. We corrected per your feedback on page 18.*

**Q6.** Conclusion part: authors should focus on modifiable risk factors: such as lifestyle changing (dietary habit, declining options of overweight and obesity) and behavioural changes. Because risk factors in your study were almost modifiable risks except gender.

**Answer for Q6.** *We appreciate for your invaluable comment. We included the feedback in the conclusion part on page 3 and 22.*

**Introduction Part**

The authors would benefit from including the diabetes trends observed in previous studies conducted in the Sidama region.

**Q1.** Before mentioning the objective of their study, it is crucial to emphasize the significant gaps that the authors identified in previous research, which motivated them to address these gaps in their own study.

**Answer to Q1.** *We appreciate your feedback. In response to your comment on page 5, We have incorporated earlier national and regional research findings.*

**Materials and methods**

**Study setting part**

The study was conducted in Hawassa town, Ethiopia. Modify it to: “The study was conducted in Hawassa town, Sidama regional state, Southern Ethiopia”

**Answer.** *We appreciate for the comment. We included "Sidama regional state, Southern Ethiopia" per the comment on page 6.*

**Answer**
Inclusion and exclusion part
Kindly provide clarification on the cases of previously diagnosed and known diabetes, as there is no information regarding them in the inclusion/exclusion

**Answer.** *Thank you for the comment. Previously diagnosed and known diabetic patients were included in the study. Please see in the inclusion and exclusion part on page 6.*

**Sample size determination part**

**Q2.** Why did the authors choose to rely solely on the prevalence rate when determining the sample size, given that their objective was to determine both prevalence and risk factors? Why didn't the authors attempt to determine the sample size using risk factors? It is advisable to calculate the sample size using both the prevalence rate and risk factors, and then select the larger sample size as the final sample size by comparing the sample sizes calculated using both methods.
Study variables part.

**Answer Q2.** *We appreciate for the feedback to consider and compare sample size determination for various associated factors. Accordingly, it was calculated as shown on table , on page 8.*

**Q3.** Why the economic/monthly income status of the study subjects was not considered as an independent factor?

**Answer for Q3.** *We appreciate your feedback. Since this research is self-sponsored, it would have required a significant investment of resources to incorporate all variables, so we only took a few of the WHO STEP surveillance guideline variables into consideration.*

**Q4.** Why the study did not take into account independent factors like comorbid health conditions, hypertension, or the use of medications for comorbid health problems among the study subjects?

**Answer for Q4.** *We appreciate your comment. We included the analysis findings of hypertension on page* *17.*

**Definitions part**

Body Mass Index (BMI): A standard cut-offs limit calculated using the formula Kg/m2 to assess an individual’s fat. Please! Correct it to: “Body Mass Index (BMI): A standard cut-offs limit calculated using the formula Kg/m2 to assess an individual’s fat

**Answer:** *We appreciate your comment. We corrected and incorporated Kg/m2 in the manuscript where necessary.*

**Data collection part**

A standard glucometer (Auto-cad) used for fasting blood test following overnight fasting prior to testing.
**Q5.** is it auto code or auto-cad?

**Answer for Q5.** *Thank you for the comment. It is auto-code. We corrected and incorporated in the manuscript accordingly.*

“The data collection period was from September, 2023 to November, 2023”.
Please! Remove this redundant sentence because once it was mentioned in the study period part.

**Answer:** *We appreciate for the feedback. I corrected redundant sentences of the study period in the manuscript where necessary.*

**Q6.** How was the accuracy of the glucometer instrument checked at first and at regular intervals during data collection?

**Answer for Q6.** *We appreciate your feedback. Calibration was done using glucometer code key both before the blood test and on a frequent basis during the data collection period.*

**Q7.** The management of data quality remains unclear as there is no information provided on how it was addressed?

**Answer for Q7.** *Data was submitted to supervisors and checked for completeness and accuracy on daily base. Moreover, prior to analysis data was checked, cleaned and entered in SPSS to ensure data quality.*

**Q8)** What actions did you or your study team take for the newly diagnosed DM subjects? It is important to address this aspect as there is no information provided about these subjects in your data collection or ethical issue sections

**Answer for Q8.** *Thank you for the comment. The newly diagnosed diabetes patients were advised on lifestyle modification. Moreover, they were told to make medical follow up at the nearby health facility at regular base.*

**Results part**

Prevalence of diabetes

**Q9.** Out of the total prevalence rate of 14.4%, what is the number of newly diagnosed cases of DM and how many are cases of previously diagnosed and known DM?

**Answer for Q9.** *The number of newly diagnosed DM patients were 101 (63.1%). The number of known DM cases were 59 (36.9%)*

**Q10.** what is the prevalence rate of prediabetes in your study?

*It is preferable to provide an explanation regarding the “Behavioural and biological risk factors of diabetes and its table” prior to discussing the prevalence of diabetes.
*please! Put footnote for all abbreviated words below table (table3: COR, AOR, CI)

**Answer for Q10.** *Thank you for the comments. The prevalence of pre-diabetes was 283 (26.9%) among study participants excluding known diabetes patients from the denominator. Behavioural and biological risk factors of diabetes were discussed prior to prevalence of diabetes per your comment. Likewise, Abbreviations were explained on page 20 below table 4.*

**Discussion part**

*Slightly higher than a study done in Hawassa zuria woredas (12.2%) [17]. Not slightly higher but comparable because your study confidence interval included the rate. The reference 17 reveals the prevalence of DM in Hawassa zuria is not 12.2% but 12.4%. Please check and correct it.

**Answer:** *Thank you for the comment. We corrected and changed to 12.4%, please see on page 20.*

*Also, the highest prevalence of diabetes (19.7%) and (18.4%) were reported in the age category between 50-59 years and 30-39 years, respectively. Please! Use the word higher but not highest

**Answer:** *We appreciate for the comment. We changed "highest" to "higher" per your comment. Please see on page 20.*

**Q11.** what is possible reason for this increment? Please! Mention it.
*In this particular research, the prevalence of diabetes was higher in Males than females,
20.7% versus 10.0% respectively. The finding is higher than the study done in Korea (males,
10.7% and females, 8.4%) [19] and Ethiopia (males, 9.1% and females, 8.4%) [20].
What are your possible reasons for the variations? Please describe it. Please! Use similar discussion manner in all discussion parts.

**Answer for Q11**: *Thank you for the feedback. We included possible reasons please see on page 21.*

**Reviewer #2:** I thank the editor for his/her invitation and the authors for their efforts to come up with this interesting scientific report. I suggest and inquire the authors to make their report more robust and readable.
“…risk factors…” in the title should be replaced by ‘associated factor’.

**Answer:** *We appreciate for the comment. We replaced risk factor by associated factor in the title page as per the comment please see on page 1.*

The authors included the population between ages 20 to 69? What were your rationale behind the inclusion this particular population?

**Answer**: *The population 20 to 69 years were included in this study for proportional sample size allocation for the age category based on the national statistical report.*

The authors stressed description of type-2 diabetes in the introduction section. Was their study only assessed the prevalence of type-2 diabetes?

**Answer**: *Thank you for the comment. The study focused primarily prevalence of diabetes and associated factors among adult population in Hawassa town which could be both type II and type I diabetes. Per the comment, prevalence of type I diabetes was incorporated in the introduction part of the manuscript please see on page 3 and 4.*

The introduction section did not show your depth of knowledge about the risk factors of diabetes, your second specific objective.

**Answer:** *Thank you for your comment. We have incorporated risk factors associated with diabetes in the introduction section in detail per your comment please see on page 4 and 5****.***

The study setting is not detail. The number of sub-cities and the estimated number of house-holds would be included.

**Answer.** *Thank you for the feedback. We included the number of sub-cities, villages (kebeles) and estimated households in the town please see on page 6 .*

The authors’ excluded pregnant women, physical disabilities, terminally sick and individual with mental illness. Why did you exclude all physical disabled and mentally ill individuals? It seems like discrimination. You don’t think people with physical disability and mental problems develop diabetes in their lives? Also, known diabetic women could get pregnant and still might be excluded from this study.

**Answer**. *We appreciate for the comments. It is apparent that all individuals could be at risk of having diabetes if exposed to risk factors. However, pregnant women were excluded in this study because they might have gestational diabetes which may result in prevalence bias. Furthermore, while taking weight still would reflect bias on body mass index findings. Likewise, Patients with mental illness were excluded because they were not eligible to provide correct information during interview. Also, physical disabilities need further care with special instrument support while taking multiple measurements. Due to these facts, they were not included in this study.*

How did you manage when you got more than one eligible individuals (aged 20 to 69) in the selected house-holds?

**Answer.** *We used kish method (random selection of study subjects from eligible age groups at household level) to select a single individual when there were more than one eligible individuals in one household.*

Under “variables” subheading, it would better to say ‘prevalence of diabetes’
Term definitions would be cited.

**Answer.** *Thank you for the comment. We included and corrected as prevalence of diabetes per the comment please see on page 8.*

“Currently tobacco smoking” this would be time bounded. What if an individual had been smoked until the last month prior to your survey?

**Answer.** *Thank you for the comment. All level of smoking status including past smoking history were included in the study as shown on Table 3, please see on page 16. However, in this particular study “current smoking" fulfils criteria for multi variable analysis.*

The authors measured the BMI of the participants but they did not detail the measurement instrument and procedures as well as the instrument’s calibers. How did you manage waste disposal throughout the study period?

**Answer:** *Thank you for the comments. Measurements were taken using a portable digital weight scale and height measuring instrument (Seca). Daily calibration was carried out with a standard kilogram weight. In addition, the instrument was positioned nearer a wall on a level floor. The device was tested for satisfactory operation, pointing at zero reading, before any data was collected. In addition, study participants were instructed to remove overcoats and jackets, stand erect on a beam facing the data collectors, and go barefoot. The correct protocol for disposing of waste was adhered to when gathering the data. Waste materials were collected in plastic bags and disposed of at the neighboring health facilities' incinerators for burning.*

*.*
Authors did not mention the data quality control measures they were applied. Did you check the tool’s validity? How did you ensure whether the subjects fasted over night? At what time in the morning you tested their blood sugar? If you executed it before the subject’s breakfast, you could not test the blood sugar of that all participants within two months. This issue needs explanation.

**Answer:** *I appreciate your feedback. Experts with extensive experience evaluated the tool's validity, and questions that were deemed unclear were adjusted accordingly. Additionally, on a pre-test, 5% of the sample size was examined. Because the questioner measured what it was supposed to measure, it was valid. We tested before the study participants eat their breakfast.* *Additionally, data collectors strongly recommended fasting for the entire night. Additionally, it was often questioned if individuals had fasted before the exam. We recommended that research participants take a HgbA1c test two to three months later. However, due to resource constraints, we do not conduct testing after two to three months. Please see in the data collection section on page 9 and 10.*

The authors stated that they obtained a written informed consent from the study subjects. So how did they obtained the consent from 173 uneducated (they said “illiterate” that is also uncomfortable name) participants?

**Answer.** *Thank you for the comment. Study subjects who were unable to read and write gave their consent with their finger print after the consent form was read and explained. Please, see in ethical consideration section on page 12.*

It would be better if table 3 was not congested. To do so, the p-values of the bivariable analysis could be omitted or included in the text narration. The NA variables in this table also would not be included. If they did not show association in the bivariable analysis why did you included them in this table?

Answer: *Thank you for the feedback. We removed all the p values for both bi variable and multivariable analysis. Also, variables with not candidate for multi variable analysis were removed per your invaluable comment. See table 4. Kindly be informed that name table 3 is changed to table 4.
The authors did not discuss all their findings.*

Answer: *Thank you for your feedback. All the prevalence of diabetes mellitus and all variables having association with dependent variable were discussed Please see on page 20,21 and 22.*

The authors would state the shortcomings of their study.

**Answer**: *This study had a number of limitations. Interviewees might had eaten breakfast prior to fasting blood test. However, before testing, data collectors have frequently questioned respondents about whether or not they had eaten breakfast in an effort to close this gap. Furthermore, Pregnant women could affect body mass index values. To minimize such bias, data collectors were able to question respondents about the status of pregnancy, menstrual cycle and amenorrhea prior to taking weight. Please see on page 22.*
