# Supplementary material for: Prevalence and associated factors of diabetes among adult populations of Hawassa town, southern Ethiopia: A community based cross-sectional study
Source: PLoS One. 2025 Jan 30;20(1):e0318081. doi: 10.1371/journal.pone.0318081 (PMC11781642; doi:10.1371/journal.pone.0318081)
Supplement: Attachment — Submitted filename: Response_to_reviewers_auresp_2.docx [file pone.0318081.s002.docx]

**Dear Editor**

We appreciate you looking over our manuscript and providing us with this valuable input. We made changes to the manuscript in response to the comments, and one of the attached manuscript files shows the highlighted changes. Point by point, we offer our answer, the questions, and our response as follows:

**Comments on Journal requirements:**

**Title page**

**C1**. Title: Please! Correct it to “Prevalence and associated factors of diabetes among adult populations of Hawassa town, southern Ethiopia. A community based cross-sectional study”

**Answer for C1**. *Thank you for this important comments. We have included in the title the word “southern” per comments on page 1*.

**Abstract part**

**C2**. Background
Therefore, the aim of this study was to investigate the magnitude and preventable risk factors of diabetes among adult populations in Hawassa town, Ethiopia
But in your included non-modifiable risks like sex please modify it to “Therefore, the aim of this study was to investigate the magnitude and risk factors of diabetes among adult populations in Hawassa town, southern Ethiopia” “Preventable risk factors” Please! Correct it to “modifiable risk factors” because modifiable risk factors" is generally the better term to use when discussing factors that can be changed or controlled to reduce the risk of disease or adverse outcomes.

**Answer for C2.** *Thank you for your feedback. We corrected* “*Preventable risk factors*” *to* “*Modifiable risk factors*” *and also included the word southern per your comments on page 2.*

**C3**. A community based cross-sectional study was conducted from September, 2023 to November, 2023 among adult populations” avoid redundant words “Hawassa town, Ethiopia” because the words described in the abstract introduction section.

**Answer for C3.** *Thank you for your comment. As per the comment removed redundant words “Hawassa town, Ethiopia*” *on page 2.*

C4. Methods If you are interested, you can use this "An interviewer-administered questionnaire was used to collect data. Additionally, participants were also instructed to fast overnight, after which the standard fasting blood sugar test was conducted. A binary logistic regression model was fitted to identify independent predictors of diabetes."

**Answer for C4.** *Thank you for the comment. We re-written as follows:* Additionally, participants were also instructed to fast overnight, after which the standard fasting blood glucose test was conducted. A binary logistic regression model was fitted to identify independent predictors of diabetes." per your comment *on page 2.*

**C5.** Conclusion If you are comfortable, you can use this “This study identified a high prevalence of diabetes among adults in Hawassa, driven by various risk factors. This presents an opportunity to mitigate diabetes risk through public health measures, including avoiding khat chewing, promoting healthy diets, managing overweight and obesity, implementing community-based screening, enhancing health literacy, and integrating health information into daily life.”

**Answer for C5**. *Thank you for the comment. We corrected per your feedback on page 3.*

**C6:** Also, the magnitude of khat chewing in Hossana, Ethiopia reported at 58%, of those khat chewers, 75.2% and 24.7% were men and women, respectively [19]. It would be more informative if the authors presented data on the prevalence of diabetes/prediabetes and examined the association between khat chewing and the increased prevalence of these conditions. The above statement only addresses the magnitude of khat chewing without linking it to blood sugar levels or diabetes risk. You can use the following 2 or 3 literatures from the following 3 links ".
1. <https://pmc.ncbi.nlm.nih.gov/articles/PMC7024885/pdf/dmso-13-307.pdf>
2. file:///C:/Users/admin/Downloads/Prevalence_and_associated_risk_factors_of_pre-diab.pdf
3. <https://www.dovepress.com/article/download/71642>

**Answer for Q6.** *We appreciate for your comment. We included the two references per your comment on page 5.*

**C7.** Introduction part last sentence: please! Modify it to “A previous study conducted in Hawassa town was facility-based and mainly focused on individuals seeking medical care. In contrast, this study was aimed to determine the prevalence of diabetes and risk factors among adults through a community-based survey in Hawassa town, southern Ethiopia”

**Answer to C7.** *We appreciate your feedback. We modified it as per your comment on page 5 & 6.*

**Materials and methods part**

**Study setting part**

**C8.** There are also 81,523 households in the town

**Answer for C8.** *We appreciate for the comment. We included the word "the" per the comment on page 6.*

**Study design and period**

**C9.** You mentioned only the study design but not period and please! Correct it to “A community based cross-sectional study design was employed from September, 2023 to November, 2023 in Hawassa town, southern Ethiopia.”

**Answer to C9:** *Thank you for your comment. We have included both the study design and period per comment on page 6.*

**Definition of terms part**

**C10.** Pls! Correct it to “Body Mass Index (BMI): A standard cut-offs limit calculated using the formula Kg/m2 to assess an individual’s body mass and classified as underweight (<18.5kg/m²), normal weight (18.5–24.9kg/m²), overweight (25–29.9kg/m²), and obesity (≥30kg/m²). Reference: A healthy lifestyle - WHO recommendations. <https://www.who.int/europe/news-room/fact-sheets/item/a-healthy-lifestyle---who-recommendations>

**Answer for C10.** *We appreciate for the feedback. We corrected the definitions per the comments on page 8.*

**C11.** Diabetes: Pls! correct it to “It is defined as a fasting blood glucose level of ≥126 mg/dL (or ≥7.0 mmol/L), a self-reported diagnosis of diabetes, or the use of oral or injectable hypoglycemic agents."

**Answer for C11.** *We appreciate your feedback. We have corrected as per the comment on page 8.*

**C12.** Fasting blood test: please! Correct it to “Fasting blood glucose test”

**Answer for C12.** *We thank you for your comment. We corrected the word* “*Fasting blood sugar test*” *to “Fasting blood glucose test*” *on page 9, 10, 11& 16.*

**C13**. Please! Minimize Ethical consideration as much as possible.

**Answer for C13:** *We appreciate your comment. We minimized ethical consideration as follows: The university of South Africa College of Human Science Research Ethics Committee approved and granted final ethics clearance to undertake the study (CREC Reference # 13652133_CREC_CHS_2023 Dated 31 July 2023). Written informed consent was obtained from all study participants. See on page 12.*

**Discussion part**

("Please refine the Discussion section to make it more insightful.") Use the below paragraph as a sample
**C14.** "The finding is consistent with previous community-based studies conducted in southern Ethiopia, such as the 14.7% prevalence in the ostracized Menja community [25] and 12.4% in the Hawassa Zuria Woreda [29]." In contrast, the finding is higher than those of previous studies conducted in other countries, such as Botswana (9.3%), Zanzibar (4.4%), and Thailand (9.9%) [21, 22, 31]."

**Answer for C14.** *Thank you for the comment. We have re-written per the comment in the discussion part to make it more insightful on page 19 & 20.*

**Conclusion part**

If you are interested use this “In conclusion, this study identified a noticeable prevalence of diabetes and its association with modifiable risk factors among the adult population of Hawassa town. The evidence-based risk factors associated with diabetes highlight the need for urgent public health interventions, particularly community-level screening programs aimed at early detection and [prevention.in](http://prevention.in/) addition, the findings emphasize the importance of addressing behavioral practices, such as khat chewing, and promoting lifestyle modifications, including dietary changes and the reduction of overweight and obesity. Regular health check-ups and follow-up care for individuals with diabetes are also crucial. Moreover, risk communication strategies for diabetes should be integrated into public health campaigns to raise awareness. Lastly, these findings could serve as a baseline for future studies focused on operational study related to diabetes management and prevention.”

**Answer for conclusion part.** *We appreciate your feedback. We have re-written the conclusion part per your comment on page 21.*
